# Modeling and Position Control Simulation Research on Shape Memory Alloy Spring Actuator

**DOI:** 10.3390/mi13020178

**Published:** 2022-01-25

**Authors:** Bingshan Hu, Fengchen Liu, Binghao Mao, Zhiwei Chen, Hongliu Yu

**Affiliations:** 1 Institute of Rehabilitation Engineering and Technology, University of Shanghai for Science and Technology, Shanghai 200093, China; 213332681@st.usst.edu.cn (F.L.); 213332782@st.usst.edu.cn (B.M.); 213332784@st.usst.edu.cn (Z.C.); yhl98@hotmail.com (H.Y.); 2 Shanghai Engineering Research Center of Assistive Devices, Shanghai 200093, China

**Keywords:** shape memory alloy, spring, model, simulation

## Abstract

The shape memory alloy (SMA) actuator is widely used in aerospace, medical and robot fields because of its advantages of low driving voltage, large driving force, no noise and high-power–weight ratio. Therefore, it is of great significance to establish the theoretical model of the SMA actuator and analyze the driving characteristics of the SMA actuator. On the basis of summarizing the constitutive model of the shape memory alloy spring, the phase transformation dynamics model of SMA including the minor hysteresis loop is established using the Duhem model in this paper, and the theoretical models of the bias and differential SMA spring actuator are established. At the same time, a PID position controller including anti-saturation and anti-overheating functions is proposed to control the position of the SMA actuator. Finally, the position control simulation model of the SMA spring actuator is established and simulated. Simulation results show that the position of the SMA actuator can be well controlled by using the model and control method established in this paper.

## 1. Introduction

With the continuous development of intelligent materials and micro electromechanical systems, new actuator technology is also developing rapidly. At present, new actuators include the piezoelectric actuator, shape memory alloy, ionic polymer actuator, magnetostriction actuator, electrostatic actuator, bimetallic actuator and so on [1]. The SMA actuator uses the shape memory effect and the mechanical characteristics of the weak martensite phase at room temperature and the strong austenite phase at high temperatures to perform external work [2]. Compared with other new actuators, the SMA actuator is widely used because of its advantages of a low driving voltage, large driving force, no noise and high power-to-weight ratio. The experimental results given in reference [3] show that when the weight is less than 100 g, the power-to-weight ratio of SMA is higher than any commonly used actuator; reference [4] analyzes and compares three kinds of thermal actuators: The SMA actuator, bimetallic actuator and wax actuator, and points out that the advantages of the SMA actuator are the unrestricted output action direction, high power-to-weight ratio and large output force and output displacement. The SMA actuator is currently used in many fields such as automobiles, aerospace, medical apparatus and micro robots [5]. S. Seelecke and I. Muller gave an overview of shape memory alloy applications in the field of smart structure actuation [6] and S. Seelecke reinterpreted the one-dimensional SMA model. The model is based on a continuous, multi-well free energy that governs the phase change at a mesoscopic material scale, and the model offers extended capabilities and a simpler formulation [7]. Coiled SMA actuators can be used to drive a 3D-printing manipulator [8]. Antonia Weirich and Bernd Kuhlenkötter have pointed out the possibility and correlative critical requirements for the implementation of SMA actuators in aircraft interiors [9]. Nisha Bhatt et al. use the existing theoretical model to evaluate the force capabilities of an SMA spring and point out the great potential of the SMA spring actuator in the application of micro robots [10]. The common forms are wire and spring. The SMA wire actuator refers to the actuator using SMA wire as the driving element. When the driving element is changed from SMA wire to a spring wound by SMA wire, it is called a spring actuator. The SMA spring actuator can produce displacement greater than 100% of its original length, which means it has a wider range of applications than the SMA wire actuator [11]. For example, Seong Jun Park et al. proposed a new SMA spring-based fabric muscle (SFM) and verified the availability of SFM as a soft actuator through performance evaluation [12]. An SMA-based spring actuated gripper (SAG) was designed and developed for facilitating minimally invasive surgeries (MIS) [13]. Using the large deformation characteristics of the SMA spring and the eccentric rotating mechanism, a compact new motor that can rotate continuously can be designed [14]. Ranjith Pillai R designed a parallel platform robotic system based on the large strain characteristics of the SMA spring [15]. The SMA spring actuator is also applied to various bionic robots, such as the worm robot [16], micro bionic fish robot [17,18] and jumping robot [19]. Ryan M. Bena used a high-frequency SMA bending actuator in a steerable robot, which caused the micro insect robot SMARTI to show good mobility [20].

In order to study the properties of the SMA spring actuator, it is necessary to establish the theoretical model of the SMA spring actuator. Due to the martensitic transformation or reverse martensitic transformation of SMA material when the temperature and stress change, the relationship between the output force and output displacement of the SMA actuator is nonlinear, which brings great difficulties to the description of its constitutive relationship. The constitutive relationship of SMA refers to the change relationship between stress, strain and temperature during phase transformation. In order to describe the constitutive relationship of SMA materials, scholars from various countries have constructed different types of constitutive relationships from different angles. R. Cortez Vega attempted to use a hybrid dynamic model to describe the relationship between temperature, elongation and inner force in a spring SMA actuator [21]. Pierre-Antoine Gédouin paid special attention to the R-phase shape memory alloy helical spring actuator and put forward its corresponding simplified model [22].

The macroscopic phenomenological model is the most widely used in engineering [23]. In 1986, Tanaka established the incremental constitutive relationship of SMA material based on the Helmholtz free energy by using Clausius Duhem inequality [24], and then Liang and Rogers replaced Tanka’s exponential phase transformation evolution equation with a cosine phase transformation evolution equation, and assumed that the elastic modulus, phase transformation modulus and thermoelastic modulus of SMA material were constants to obtain a full-quantity unique constitutive equation [25]. In terms of the theoretical model of the SMA spring, Tobushi and Tanaka proposed the relationship between load and deformation of the SMA coil spring based on the stress–strain-temperature model of SMA material proposed by Tanaka [26]. Based on the thermodynamic constitutive model proposed by Liang and Rogers, they established the force displacement relationship of the SMA coil spring [27]. Aguiar et al. used the SMA material constitutive model proposed by Paiva et al. to study the hyperelasticity and shape memory effects of SMA coil springs [28,29]. Enemark used the improved Brinson model to carry out theoretical analysis and experimental verification on the mechanical properties of the SMA coil spring [30].

When an SMA spring is heated, if the initial temperature is lower than the start temperature of the austenite transformation and the final temperature is higher than the end temperature of the austenite transformation, the martensite content in the SMA material decreases from 1 to 0. When the SMA material is cooled, if the initial temperature is higher than the start temperature of the martensitic transformation and the final temperature is lower than the end temperature of the martensitic transformation, the martensite content in SMA material increases from 0 to 1. In the above transformation process, complete martensite or austenite transformation occurs in the SMA material. At this time, the hysteresis loop formed by the relationship curve between the martensite content and temperature is called the main hysteresis loop. However, in the process of practical application, SMA materials do not have complete martensite or austenite transformation in many cases. At this time, the hysteresis loop formed by the relationship curve between the martensite content and temperature is called minor hysteresis loop. Currently, most existing SMA spring models do not consider the occurrence of an incomplete martensitic transformation in SMA material under repeated loading [11]. G. Rizzello and S. Seelecke present an irreversible port-Hamiltonian model for describing the hysteresis in SMA wire actuators. The model can quantify the energy performance of SMA wires during non-isothermal driving and evaluate the thermodynamic consistency of the system based on irreversible entropy generation [31]. At present, the phenomenological model is generally used to describe the hysteresis characteristics, and the Preisach model is the most widely used in the phenomenological model. For example, the literature [32] uses the Preisach model to describe the hysteresis characteristics of SMA materials, but the calculation of the weight function in the Preisach model is quite difficult, and the Duhem model can solve the above problems because it uses differential equations to describe the hysteresis loop [33]. In this paper, the Duhem model is used to establish the phase transformation dynamic model of the SMA spring under an incomplete phase transformation. On this basis, according to the constitutive model of the SMA material, the theoretical models of bias and differential SMA spring actuators are established and simulated.

The following contents of this paper are organized as follows: The second section briefly introduces the driving principle of the SMA spring actuator. In the third section, the modeling process of the SMA spring actuator is introduced, including the constitutive model of the shape memory alloy, the phase transformation dynamic model, the spring thermodynamic model and the SMA spring actuator model. In the fourth section, the simulation model is established according to the theoretical model, and the position control characteristics of bias and differential SMA spring actuators are simulated and analyzed respectively, and their advantages and disadvantages are compared. The fifth section gives the conclusion.

## 2. Working Principle of Shape Memory Alloy Spring Actuator

According to whether the SMA spring actuator can automatically realize reciprocating motion, the actuator can be divided into a one-way actuator or a two-way actuator. The one-way SMA actuator is realized by using the one-way memory effect of SMA. It can be restored to the ready state only under the intervention of an external force. The two-way SMA actuator refers to an SMA actuator that can automatically realize reciprocating motion without external force intervention. The most direct way to realize the two-way drive of an SMA actuator is to use the two-way shape memory effect of SMA. In addition, it can also be obtained by adding a bias element to the SMA element with a one-way memory effect. When the bias element is an ordinary spring, a weight or some other non-SMA element, it is called a bias actuator. When the deflection element is another SMA element, it is called a differential actuator.

**Figure 1:**
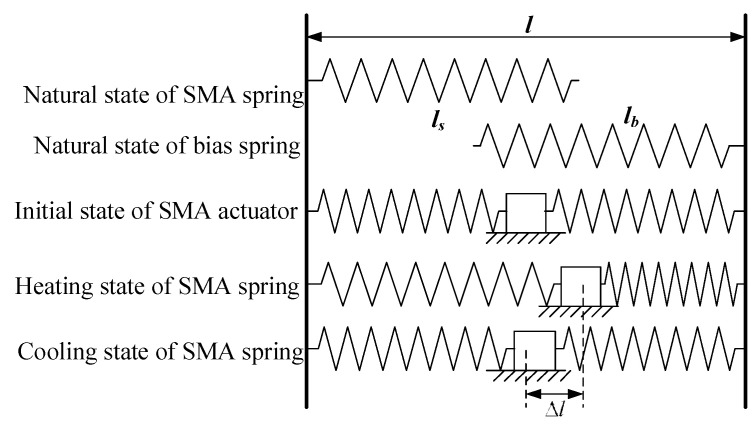
Analysis of driving principle of bias SMA spring actuator.

The working principle of bias and differential SMA spring actuators is described in Figure 1, in which the SMA spring and the bias spring are compressed in the initial state. After heating, the SMA spring extends, the elastic coefficient increases, the bias spring shortens under the action of its thrust and the load connected to the two springs moves to the right. When the SMA spring cools, because the thrust of the bias spring is greater than that of the SMA spring, the SMA spring gradually returns to the initial position under the action of the deformation restoring force of the bias spring. Different from the bias type, the differential actuator uses another SMA spring instead of the bias spring. In the initial state, both SMA springs are compressed. When one SMA spring is energized and heated, the other spring is not energized, and the load moves to one side. When the two SMA springs are heated and cooled alternately, the load can move in two directions. Compared with the bias type, the differential actuator can be driven in both directions, and the process of returning to the initial position can be actively controlled.

## 3. Modeling of Shape Memory Alloy Spring Actuator

This section introduces the constitutive model, phase transformation kinetics model and SMA thermodynamics model of shape memory alloy materials. On this basis, the theoretical models of bias and differential SMA spring actuators are established, which lays a foundation for the theoretical analysis of electrothermal drive characteristics of SMA actuators.

### 3.1. Shape Memory Alloy Spring Model

#### 3.1.1. Constitutive Model of Shape Memory Alloy

Based on Helmholtz free energy, Tanaka established the incremental constitutive relationship of SMA material using Clausius–Duhem inequality. In this paper, Tanaka’s SMA material constitutive model was adopted. In this model, the stress (σ) of the SMA wire is a function of the strain (ε), temperature (T) and austenite content ($\xi_{a}$) and its relationship is

(1) $$\dot{\sigma }=D_{sma}\dot{\varepsilon }+\Theta \dot{T}+\Omega \dot{\xi }$$

(2) $$D_{sma}=(1-\xi_{a})D_{M}+\xi_{a}D_{A}$$

In Equations (1) and (2), $D_{sma}$ is the elastic modulus of the SMA material and $D_{A}$ and $D_{M}$ are the elastic modulus of austenite and martensite of SMA materials, respectively; Θ is the thermoelastic modulus; Ω is the phase transformation modulus, $\Omega =-D_{sma}\varepsilon_{L}$, and $\varepsilon_{L}$ is the maximum recoverable strain. In the quasi-static condition, we integrate Equation (1) to obtain

(3) $$\sigma -\sigma_{0}=D_{sma}(\varepsilon -\varepsilon_{0})+\Theta (T-T_{0})+\Omega (\xi_{m}-\xi_{0})$$

Equation (3) reflects the constitutive equation of SMA wire under one dimension tension compression status, but only shear stress τ and shear strain γ exist in the deformation process of the SMA spring. In order to apply the constitutive relation of the SMA material, the equivalent stress relation in plastic mechanics $\sigma =\sqrt{3}\tau$ is introduced, in which τ is shear stress. The equivalent strain relationship $\sigma =\sqrt{3}\tau$ is also introduced, where γ is shear strain and *G_sma_* is the shear elastic modulus. According to the constitutive relationship of the SMA material, the constitutive relationship of the SMA material under a shear condition is [34]

(4) $$\tau -\tau_{0}=G_{sma}(\gamma -\gamma_{0})+\frac{\Theta }{\sqrt{3}}(T-T_{0})+\frac{\Omega }{\sqrt{3}}(\xi_{m}-\xi_{0})$$

where the shear elastic modulus *G_sma_* is

(5) $$G_{sma}=\frac{D_{sma}}{2(1+\mu_{sma})}$$

In Equation (5), $\mu_{sma}$ is Poisson’s ratio of the SMA material. Assuming that the force generated by the SMA spring after heating is *F_sma_*, the displacement is *y*, and the number of effective turns, pitch diameter and wire diameter are *n*, *D* and *d*, respectively, and the shear stress τ and shear strain γ in the SMA spring, respectively, are

(6) $$\tau =\frac{8F_{sma}D}{\pi d^{3}},\;\gamma =\frac{8F_{sma}D}{G_{sma}\pi d^{3}}$$

The relationship between the spring output displacement *y* and shear strain γ is

(7) $$y=\frac{\pi nD^{2}}{d}\gamma$$

From Equation (4), we can obtain

(8) $$\gamma =\frac{\tau -\tau_{0}}{G_{sma}}-\frac{\Theta }{\sqrt{3}G_{sma}}(T-T_{0})-\frac{\Omega }{\sqrt{3}G_{sma}}(\xi_{m}-\xi_{0})+\gamma_{0}$$

If we substitute the above equation into Equation (7) and set the stress and strain in the SMA spring at the initial moment as 0, then

(9) $$y=\frac{\pi nD^{2}}{dG_{sma}}\tau -\frac{\pi nD^{2}}{dG_{sma}}\frac{\Theta }{\sqrt{3}}(T-T_{0})-\frac{\pi nD^{2}}{dG_{sma}}\frac{\Omega }{\sqrt{3}}(\xi -\xi_{0})$$

The relation between the output force (*F_sma_*), output displacement (y), martensite content ($\xi_{m}$) and temperature (*T*) of the general SMA spring obtained by combining and simplifying Equations (4) and (9) is

(10) $$F_{sma}=\frac{d^{4}G_{sma}}{8nD^{3}}y+\frac{\pi d^{3}}{8D}\frac{\Theta }{\sqrt{3}}(T-T_{0})+\frac{\pi d^{3}}{8D}\frac{\Omega }{\sqrt{3}}(\xi_{m}-\xi_{0})$$

#### 3.1.2. The Phase Transformation Kinetics Model of Shape Memory Alloy

The austenite content $\xi_{a}$ is related to the temperature *T* and stress σ during the phase transformation. Liang replaces Tanka’s exponential phase transformation evolution equation with the cosine phase transformation evolution equation, and assumes that the elastic modulus, phase transformation modulus and thermoelastic modulus of the SMA material are constants, and finally obtains the one-dimensional constitutive equation of the full quantity type. This model can describe the main hysteresis loop well, but it cannot describe the minor hysteresis loop. The Duhem model can solve the above problems by using differential equations to describe the hysteresis loop. Therefore, this paper adopts the Duhem model to establish the phase transformation kinetics model of the SMA material including the minor hysteresis loop [26].

The Duhem model puts forward that the output characteristics change only when the hysteresis input direction changes. We set the input variable of the hysteresis system as the temperature *T* of the SMA material, and the output variable as the austenite content *ξ_a_*. Ignoring the influence of the stress change of the SMA material on the phase transformation temperature, the relationship of *ξ_a_-T* in the SMA material can be expressed by the Duhem model as

(11) $$\frac{d\xi_{a}}{dt}=\{\begin{array}{c} g_{+}(T(t),\xi_{a}(t))\frac{dT}{dt}\begin{array}{cc} & \frac{dT}{dt}\geq 0 \end{array}\\ g_{-}(T(t),\xi_{a}(t))\frac{dT}{dt}\begin{array}{cc} & \frac{dT}{dt}<0 \end{array} \end{array}$$

*g_+_* and *g_−_* are slope functions of the austenite content *ξ_a_* relative to the temperature *T* when the temperature *T* increases or decreases with time *t*, respectively. As in the literature [26], this paper adopts the normal distribution function as the slope function in the Duhem model, that is

(12) $$g_{+/-}(T)=\frac{1}{\delta_{+/-}\sqrt{2\pi }}\exp(-\frac{{\left(T-\mu_{+/-}\right)}^{2}}{2\delta_{+/-}^{2}})$$

where μ and δ are the mean value and standard deviation, respectively. If we substitute Equation (12) into Equation (11), then the main hysteresis loop of the SMA material can be described by the Duhem model as

(13) $$\frac{d\xi_{a}}{dT}=\{\begin{array}{c} \frac{1}{\delta_{+}\sqrt{2\pi }}\exp(-\frac{{\left(T-\mu_{+}\right)}^{2}}{2\delta_{+}^{2}})\begin{array}{cc} & \frac{dT}{dt}\geq 0 \end{array}\\ \frac{1}{\delta_{-}\sqrt{2\pi }}\exp(-\frac{{\left(T-\mu_{-}\right)}^{2}}{2\delta_{-}^{2}})\begin{array}{cc} & \frac{dT}{dt}<0 \end{array} \end{array}$$

Austenite content *ξ_a_* in SMA can be obtained by integrating Equation (13)

(14) $$\xi_{a}{}_{+/-}=h_{+/-}(T)=\int_{-\infty }^{T}g_{+/-}(T^{\prime })dT^{\prime }=\frac{1}{2}\left[1+erf(\frac{T-\mu_{+/-}}{\sqrt{2}\delta_{+/-}})\right]$$

In Equation (14), “*erf*” represents the error function. Equations (11)–(14) describe the main hysteresis loop of the SMA phase transformation. *ξ_a_*_+_ and *ξ_a_*_−_ represent the austenite content in the SMA material at the heating and cooling stages, respectively. *h*_+_(*T*) and *h*_−_(*T*) are the austenite functions with temperature *T* as the independent variable during the heating and cooling processes.

Now, the Duhem model is used to describe the minor hysteresis loop, first assuming that the main and minor hysteresis loop have the same shape. Bekker and Brinson verified the correctness of this hypothesis using experimental data in the literature [35]. We assume that the slope function *gi*_+/−_(*T*) of the ith minor hysteresis loop is proportional to the slope function *g*_+/−_(*T*) of the main hysteresis loop, which can be written as

(15) $$g_{i+/-}(T)=n_{i+/-}g_{+/-}(T)$$

where the proportional coefficient is *n_i_*_+/−_. We set the austenite function in the cooling process of the *i*th minor hysteresis loop as *h_i_*_−_(*T*), then

(16) $$n_{i-}=\frac{g_{i-}(T)}{g_{-}(T)}=\frac{h_{i-}(T)-h_{+}(T)}{h_{-}(T)-h_{+}(T)}$$

By substituting Equation (15) into Equation (16), the slope function in the cooling process of the minor hysteresis loop can be obtained

(17) $$g_{i-}(T)=\frac{h_{i-}(T)-h_{+}(T)}{h_{-}(T)-h_{+}(T)}g_{-}(T)$$

In the main hysteresis loop, *h_i_*_−_(*T*) is *h*_−_(*T*), and the slope function *g_i_*_−_(*T*) is *g*_−_(*T*). Therefore, the slope function of the main hysteresis loop is a special case of Equation (17). In the same way as in Equations (15)–(17), the slope function *g_i_*_+_(*T*) in the heating process of the minor hysteresis loop can also be obtained, so the *ξ_a−_T* hysteresis model of the SMA material including the main and minor hysteresis loops is

(18) $$\frac{d\xi_{a}}{dT}=\{\begin{array}{c} \frac{h_{-}(T)-\xi_{a}}{h_{-}(T)-h_{+}(T)}g_{+}(T)\begin{array}{cc} & \frac{dT}{dt}\geq 0 \end{array}\\ \frac{\xi_{a}-h_{+}(T)}{h_{-}(T)-h_{+}(T)}g_{-}(T)\begin{array}{cc} & \frac{dT}{dt}<0 \end{array} \end{array}$$

The austenite content *ξ_a_* in the SMA material can be obtained by integrating Equation (18) with the temperature, where *h_+/−_*(*T*) and *g*_+/−_(*T*) can be obtained by Equations (12) and (14).

#### 3.1.3. Thermodynamic Model of Shape Memory Alloy Spring

Since the SMA actuator outputs force and displacement by temperature change, the temperature change is achieved by heating and cooling the actuator. The thermal conductivity differential equation of the SMA actuator in the process of the temperature change is

(19) $$\frac{\partial t}{\partial \tau }=\frac{h}{\rho c}(\frac{\partial^{2}t}{\partial x^{2}}+\frac{\partial^{2}t}{\partial y^{2}}+\frac{\partial^{2}t}{\partial z^{2}})+\frac{\dot{\Phi }}{\rho c}$$

where *t*, τ, *c*, *ρ*, *h* and Φ are, respectively, the temperature, time, specific heat, density, thermal conductivity and heat source intensity of the SMA material. Since the SMA model conforms to the condition of lumped parameter simplification, the thermal conductivity is independent of the temperature and coordinates inside the model, and the above thermal conductivity differential equation can be simplified as

(20) $$\frac{\partial t}{\partial \tau }=\frac{\dot{\Phi }}{\rho c}$$

In the process of electrically heating the SMA actuator, the heat source intensity generated by electric heating is

(21) $$\dot{\Phi_{t}}=\frac{\Delta \Phi_{V}}{\Delta V}=\frac{I^{2}R}{V}$$

where *R* and *V* are the internal resistance and volume of SMA material, and the negative heat source intensity generated by heat dissipation is

(22) $$\dot{\Phi_{W}}=\frac{\Delta \Phi_{W}}{\Delta V}=\frac{hA(t-t_{\infty })}{V}$$

In Equation (22), $t_{\infty }$ is the ambient temperature and A is the heat dissipation surface area of the SMA actuator. According to Equations (20)–(22), the temperature change of the SMA actuator in the electric heating process satisfies

(23) $$\rho cV\frac{dt}{d\tau }=I^{2}R-hA(t-t_{\infty })$$

The first term on the right of the above equation is absent during natural cooling of the SMA actuator. For the SMA actuator, if the driving current *I* is given, the temperature of SMA in the heating process can be determined by Equation (23) above, and the temperature is then substituted into the phase transformation kinetics model of SMA to obtain the martensite content of the SMA material at the current temperature, and then the output force and displacement of the SMA spring can be calculated according to the thermodynamics model of the SMA spring.

### 3.2. Bias Shape Memory Alloy Spring Actuator Model

The theoretical model of the bias actuator is established below. Let the length of the SMA spring and the bias spring in the natural state be ls and lb. Due to the constraint of the shell, the sum of the deformed length of the SMA spring and bias spring is l. The elastic coefficient of the bias spring is Kb. Since both the SMA spring and the bias spring are in the compression state at the initial time, let their deformations be $\Delta l_{s}^{i}$ and $\Delta l_{b}^{i}$, respectively. When the SMA spring is heated by electricity, the deformation of the SMA spring and the bias spring is $\Delta l_{s}^{h}$ and $\Delta l_{b}^{i}$. Since the length of the whole bias actuator is certain, the deformation of the two springs during heating and cooling meets the geometric relationship

(24) $$\Delta l_{b}^{i/h}=(l_{b}+l_{s})-(l+\Delta l_{s}^{i/h})$$

At the initial time, the output force of the SMA spring is equal to that of the bias spring. At this time, due to no power usage for heating, the SMA material is full of martensite, and its temperature is the ambient temperature. According to Equation (10), there is

(25) $$\frac{d^{4}G_{sma}}{8knD^{3}}\Delta l_{s}^{i}=F_{s}^{i}=F_{b}^{i}=K_{b}\Delta l_{b}^{i}$$

The deformation $\Delta l_{s}^{i}$ and $\Delta l_{b}^{i}$ of the SMA spring and the bias spring at the initial time can be obtained simultaneously by Equations (24) and (25). When the SMA spring is energized and heated, we set the output force of the SMA spring and the output force of the bias spring as $F_{s}^{h}$ and $F_{b}^{h}$, respectively, and the load force on the actuator as *F*, then

(26) $$\begin{array}{l} \frac{d^{4}G_{sma}}{8knD^{3}}\Delta l_{s}^{h}+\frac{\pi d^{3}}{8kD}\frac{\Theta }{\sqrt{3}}(T-T_{0})+\frac{\pi d^{3}}{8kD}\frac{\Omega }{\sqrt{3}}(\xi -\xi_{0})=F_{s}^{h}=\\ F_{b}^{h}+F=K_{b}\Delta l_{b}^{h} \end{array}$$

If the driving current of the SMA spring is known, the temperature of SMA at any time in the heating process can be determined according to Equation (23), and then the temperature can be substituted into the phase transformation kinetics model of SMA to obtain the martensite content of the SMA material at the current temperature, and the shear modulus of the SMA spring at this time can be obtained. Substituting the current temperature *T*, martensite content *ξ* and shear modulus Gsma of the SMA spring into Equation (26) and combining it with Equation (24), the deformation $\Delta l_{s}^{h}$ and $\Delta l_{b}^{i}$ of the SMA spring and the bias spring during heating and cooling can be obtained. During the whole driving process, the output displacement Δl of the bias SMA spring driver is

(27) $$\Delta l=\Delta l_{s}^{h}-\Delta l_{s}^{i}$$

### 3.3. Differential Shape Memory Alloy Spring Actuator Model

Similar to the establishment of the bias actuator model, supposing that two SMA springs are made of the same material, all parameters are the same, where subscripts 1 and 2 represent the parameters related to spring 1 and 2, respectively and superscripts *i*, *h* and *c* represent the initial state, heating process and cooling process parameters, respectively. We suppose the original length of both springs is *l_sma_* and the length of the whole differential actuator is *l*. Both springs are compressed in the initial state. If the compression of spring 1 is $\Delta l_{1}^{h/c/i}$, the deformation of spring 2 $\Delta l_{2}^{h/c/i}$ is

(28) $$\Delta l_{2}^{i/h/c}=2l_{sma}-\Delta l_{1}^{i/h/c}-l$$

In the initial state, the SMA material is filled with martensite and its temperature is the ambient temperature. SMA springs are regarded as ordinary springs whose elastic coefficient varies with temperature. The output force of the two SMA springs in the cooling state is equal, which can be written as

(29) $$\frac{d^{4}G_{1}^{i}}{8nD^{3}}\Delta l_{1}^{i}=F_{1}^{i}=F_{2}^{i}=\frac{d^{4}G_{2}^{i}}{8nD^{3}}\Delta l_{2}^{i}$$

The shape variables $\Delta l_{1}^{i}$ and $\Delta l_{2}^{i}$ of the SMA spring in the initial state can be obtained simultaneously by Equations (28) and (29).

When spring 1 is cooled and spring 2 is heated, the output force of spring 2 bears the deformation force of spring 1 in the case of no load at the end of the SMA actuator, so the output force of spring 2 and spring 1 satisfies this relation

(30) $$\begin{array}{l} \frac{d^{4}G_{1}^{c}}{8nD^{3}}\Delta l_{1}^{c}+\frac{\pi d^{3}}{8D}\frac{\Theta }{\sqrt{3}}(T_{{}_{1}}^{c}-T_{0})+\frac{\pi d^{3}}{8D}\frac{\Omega }{\sqrt{3}}(\xi_{{}_{1}}^{c}-\xi_{0})+F=\\ \frac{d^{4}G_{2}^{h}}{8nD^{3}}\Delta l_{2}^{h}+\frac{\pi d^{3}}{8D}\frac{\Theta }{\sqrt{3}}(T_{{}_{2}}^{h}-T_{0})+\frac{\pi d^{3}}{8D}\frac{\Omega }{\sqrt{3}}(\xi_{{}_{2}}^{h}-\xi_{0}) \end{array}$$

When spring 1 is heated and spring 2 is cooled, the output forces of spring 1 and spring 2 satisfy the relationship

(31) $$\begin{array}{l} \frac{d^{4}G_{1}^{h}}{8nD^{3}}\Delta l_{1}^{h}+\frac{\pi d^{3}}{8D}\frac{\Theta }{\sqrt{3}}(T_{{}_{1}}^{h}-T_{0})+\frac{\pi d^{3}}{8D}\frac{\Omega }{\sqrt{3}}(\xi_{{}_{1}}^{h}-\xi_{0})=\\ \frac{d^{4}G_{2}^{c}}{8nD^{3}}\Delta l_{2}^{c}+\frac{\pi d^{3}}{8D}\frac{\Theta }{\sqrt{3}}(T_{{}_{2}}^{c}-T_{0})+\frac{\pi d^{3}}{8D}\frac{\Omega }{\sqrt{3}}(\xi_{{}_{2}}^{c}-\xi_{0})+F \end{array}$$

In order to solve the position output of the differential shape memory alloy spring drive, firstly, the temperature of the SMA spring under a certain driving current is calculated according to Equation (23). Then, the martensite content in the SMA material at the current temperature is calculated according to the transformation kinetics model of the SMA material. Finally, the shear elastic modulus G1/2 of the two SMA springs at this time is calculated according to Equations (2) and (5), which is substituted into Equations (30) and (31), and $\Delta l_{1}^{h/c}$ and $\Delta l_{2}^{h/c}$ can be obtained simultaneously by Equations (28) and (30) and Equations (28) and (31), respectively. Then, the displacement of the differential SMA spring driver is

(32) $$\Delta l=\Delta l_{1}^{h/c}-\Delta l_{1}^{i}$$

## 4. Simulation Verification

### 4.1. Establishment of Position Control Simulation Model

**Figure 2:**
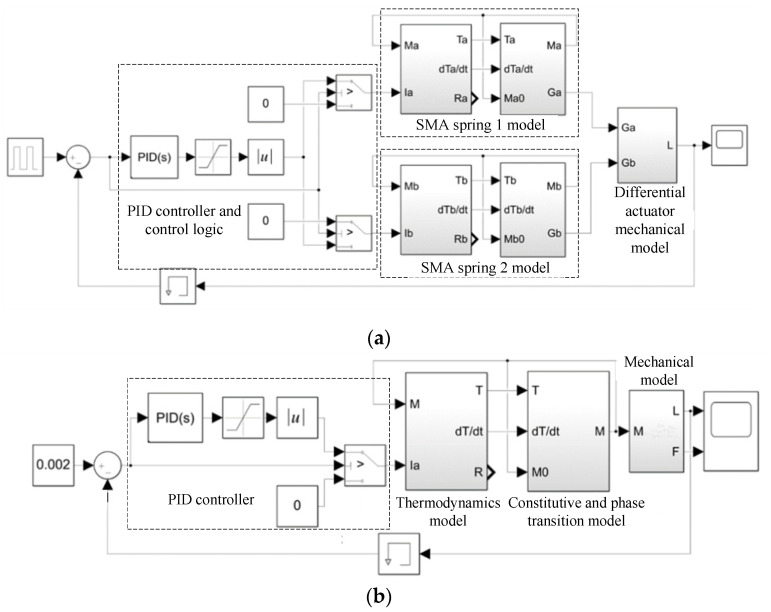
Simulation model of SMA spring actuator. (**a**) Simulation model of bias SMA spring actuator(**b**) Simulation model of differential SMA spring actuator.

**Figure 3:**
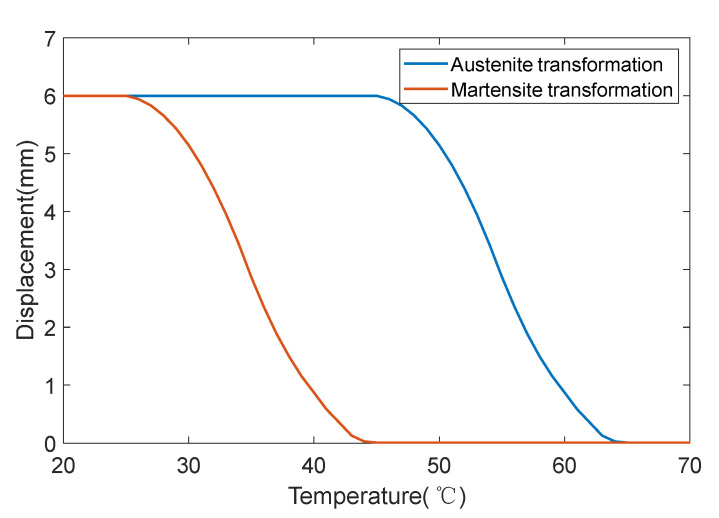
The shape memory effect of the SMA spring.

According to the above theoretical model, the MATLAB/Simulink model of the SMA spring actuator can be established in Figure 2, in which the simulation model of the bias actuator is shown in Figure 2a, and the simulation model of the differential SMA spring actuator is shown in Figure 2b. As the hysteresis characteristic of the shape memory alloy will increase the difficulty of control, Lili Meng et al. designed a closed-loop proportional integral derivative controller to compensate for the hysteresis and adjusted it via a back-propagation neural network algorithm. The results show that the controller can compensate for the hysteresis of the dielectric elastomer actuator [36]. G. Rizzello et al. proposed a controller based on the robust control theory and linear matrix inequalities, which permits one to arbitrarily shape the stiffness of the estimator while providing robust stability and performance with respect to model nonlinearities [37]. Zhenyu Shi et al. presented a newly designed SMA actuator controlled by a combination of self-sensing feedback and compact SSMA–sensing feedback, and accurate and stable motion control is demonstrated [38]. In order to realize the position control of the SMA spring actuator, this paper adopts a PID controller and adds a saturation function in the simulation to prevent the output value of PID from exceeding the maximum value of the current source. Since the SMA actuator can only heat actively and the current cannot be negative after PID, it is necessary to judge whether the feedback position of the SMA actuator exceeds the command. Once it exceeds, the SMA actuator does not heat, that is, the current passing through is 0, which can also prevent the SMA spring from overheating. The SMA actuator in this paper is designed and manufactured according to our requirements by a company. The requirements of the phase transition temperature, spring length, output force, etc., are specified. Therefore, the shape memory alloy used in this paper is considered to have passed the performance test and meet the requirements. The parameters selected in this simulation are shown in Table 1, in which the SMA material is the commercial NiTi alloy. The composition of the NiTi alloys is Ti-50.8 at.% Ni. So, the martensitic transformation systems is from B2 austenite to B19 martensite. Besides, we do not input the lattice parameters in the process of simulation. The shape memory effect of the SMA spring is shown in Figure 3.

### 4.2. Simulation of Bias Shape Memory Alloy SPRING Actuator

**Figure 4:**
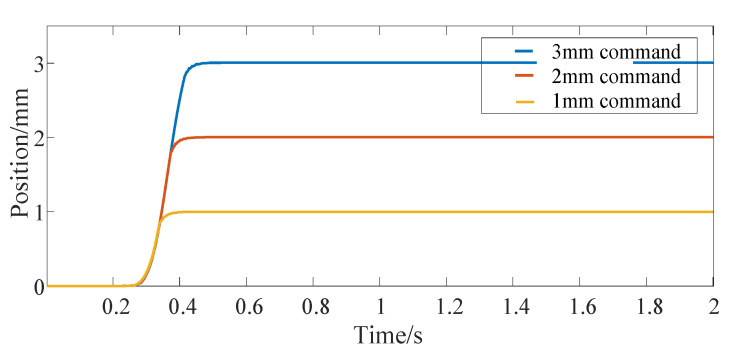
Bias actuator position step response.

**Figure 5:**
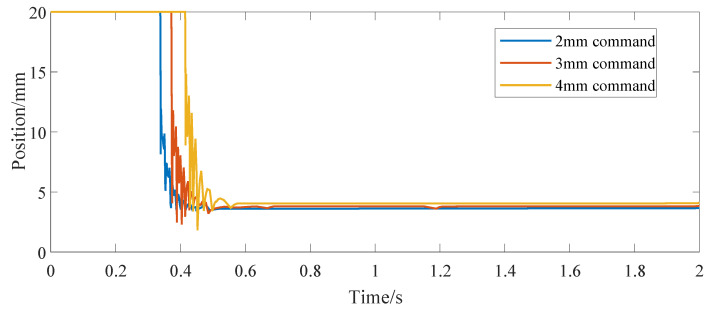
Driving current of the bias actuator for the position step command.

**Figure 6:**
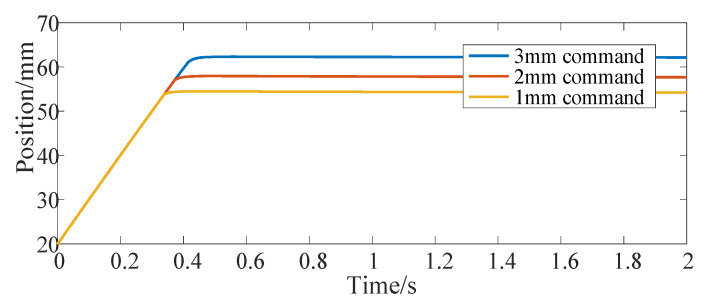
SMA spring temperature response of bias actuator for position step command.

Figure 4 shows the position response curve of the actuator after giving the step signal command. The step command is given at 0 s, and the three curves correspond to the position command of 1 mm, 2 mm and 3 mm, respectively. Figure 5 shows the driving current command of the SMA spring corresponding to them. Moreover, this paper mainly focuses on the constitutive model of SMA without in-depth study of advanced control methods, so vibration appears in Figure 5. Figure 6 shows the temperature response curve of the SMA spring. As can be seen from Figure 4, Figure 5 and Figure 6, after a given position command, due to the large deviation between the actual position of the actuator and the given command and affected by the saturation mechanism in the position controller, the SMA spring uses the maximum current provided by the current source as the driving current for heating. After the temperature of the SMA spring rises linearly for about 0.3 s, because the temperature exceeds the austenite transformation start temperature of the SMA spring, the SMA spring pushes the bias spring to start moving. After that, the displacement of the bias SMA spring actuator continues to increase. When the deviation between the actual position and the given command is small, the heating current is calculated according to the PID controller. For 1 mm position step command, the actual position of the actuator enters the steady state after 0.4 s. In order to maintain the thrust of the SMA spring, the heating current of the SMA spring is maintained at 3.6 A and the temperature is maintained at 54.5 °C. When the position command increases, the time for the position step response to reach the final steady state also increases (0.45 s at 2 mm and 0.5 s at 3 mm). At the same time, the steady-state driving current and the steady-state temperature of the SMA spring also increases (3.8 A and 57.9 °C at 2 mm and 4 A and 62.3 °C at 3 mm).

**Figure 7:**
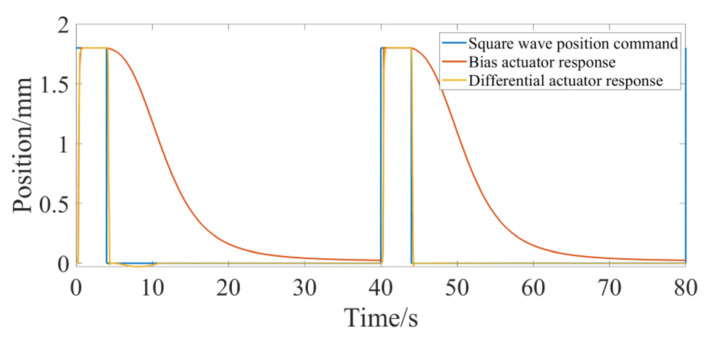
Position response of bias actuator for square wave command.

Figure 7 shows the position response of the bias actuator when the control command is a square wave signal. The amplitude of the square wave signal is 1.8 mm, its cycle is 40 s and the duty cycle is 10% (the position command is greater than 0). As can be seen from the figure, when the value of the square wave signal is high, the actual position of the SMA actuator quickly rises to the given command. When the position command is 0, the displacement of the SMA actuator decreases slowly, which takes about 30 s from 1.8 mm to about 0, because the bias actuator cannot actively control the recovery process.

### 4.3. Simulation of Differential Shape Memory Alloy Spring Actuator

**Figure 8:**
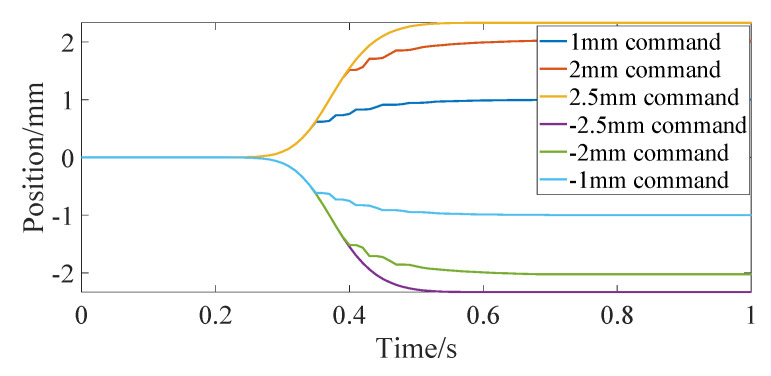
Position step response of differential actuator.

**Figure 9:**
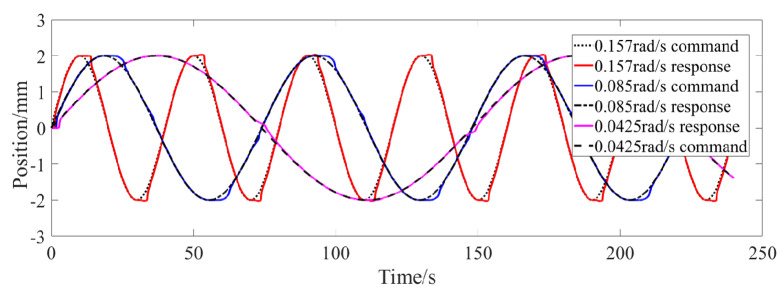
Sinusoidal position response of differential actuator.

**Figure 10:**
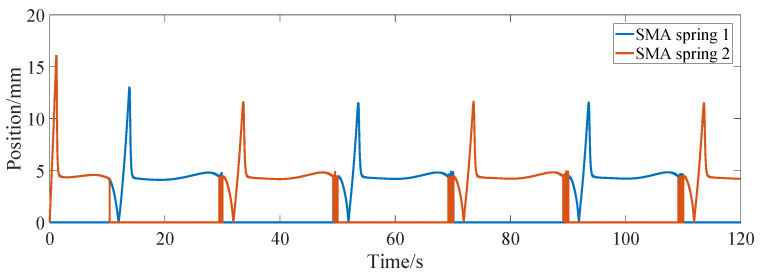
Driving current of differential actuator tracking sinusoidal position command.

**Figure 11:**
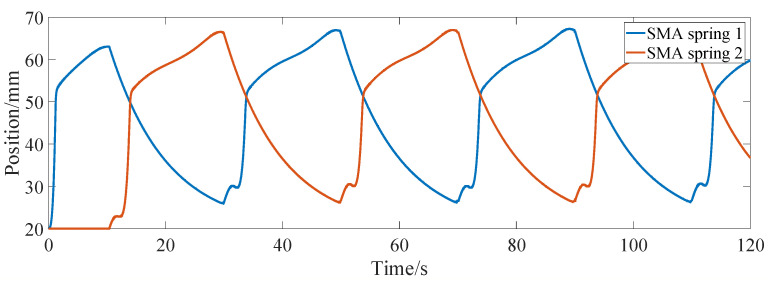
Temperature response of differential actuator tracking sinusoidal position command.

Figure 8 shows the step response curve of the differential actuator, and the corresponding commands are ±1 mm, ±2 mm and ±2.5 mm, respectively. It can be seen from the figure that the step response of the differential actuator is similar to that of the bias actuator. After about 0.5 s, the actual position of the differential actuator can reach a steady state. In order to investigate the frequency response of the differential actuator, the sinusoidal position command is given for position control simulation. Figure 9 shows the tracking of the sinusoidal command when the amplitude is 2 mm and the frequency is 0.157 rad/s, 0.085 rad/s and 0.0425 rad/s, respectively. It can be seen from the figure that the differential SMA actuator in this paper can track the above sinusoidal position command well. Near the sinusoidal command peak, there is a large deviation between the command and the actual position, mainly because the movement direction of the actuator position changes at this time, and the SMA spring driven reversely changes from heating to cooling or from cooling to heating, which has large hysteresis. Through simulation experiments, when the frequency of the sinusoidal command is greater than about 0.2 rad/s, the differential actuator in this paper cannot track the sinusoidal position command signal well. Figure 10 and Figure 11 show the SMA spring driving the current and temperature curve when tracking the sinusoidal position command with an amplitude of 2 mm and a frequency of 0.157 rad/s. As can be seen from the figure, when the sinusoidal command gradually increases from 0, the driving current of spring 1 rapidly increases to a larger value, so that the temperature of SMA spring 1 rises rapidly, and then the driving current rapidly decreases to about 4 A. When the position and moving direction of the actuator change, the driving current of spring 1 decreases to 0 and enters the cooling state, while the driving current of spring 2 increases rapidly, increasing the stiffness of spring 2. The temperature curves of the two springs are also cooled or heated alternately. It can be seen from Figure 11 that the temperature of the SMA spring varies between 27 °C and 67 °C during continuous tracking of the sinusoidal command.

Compared with the bias actuator, the step response of the differential actuator takes a relatively long time to reach the steady state, which is mainly due to the fact that the equivalent stiffness of the SMA spring of the differential actuator is slightly greater than that of the bias spring. The differential actuator adopts two SMA springs, so the differential actuator can move in both directions and has a wider range of motion when the size is the same. For the square wave command (Figure 7), compared with the bias actuator, the differential actuator can not only elongate rapidly, but also recover much faster than the bias actuator due to the active SMA spring drive in the recovery process. Due to the active control of SMA spring in the process of two-way movement, the differential SMA spring actuator has a certain dynamic tracking ability. The SMA differential actuator designed in this paper can track the sine wave with a frequency less than 0.2 rad/s. The above analysis is under the condition that the output displacement of the differential driver is relatively small. There are other problems for the differential actuator. If the driving cycle is too small, the output displacement of the differential actuator is significantly reduced, because the cooling time of the SMA spring is reduced after the cycle is reduced. When one SMA spring is heated, because the other spring is not fully cooled, the two springs reach a force balance at the new position, affecting the output displacement. Therefore, the differential actuator must have sufficient cooling time for both SMA springs.

The authors of this manuscript designed and produced a shape memory alloy actuator at an early stage, but at present, the drive control system of the actuator is still under construction, so this manuscript mainly carries out simulation research. Once the drive control system is built, experimental research will be carried out.

## 5. Conclusions

In this paper, on the basis of the analysis of the principle of a shape memory alloy spring actuator, based on the constitutive model of the shape memory material, a kinetics model of the phase transformation of the SMA material including minor hysteresis loops is established using the Duhem model, and then combined with the thermodynamic model of the SMA spring, theoretical models of bias and differential SMA spring actuators are established. This paper also designs an SMA actuator position PID controller including anti-saturation and anti-overheating functions. Based on the above theoretical model, the SMA actuator position control simulation model is established. The position control of bias and differential SMA spring actuators are simulated and analyzed, respectively. The analysis results show that the theoretical model established in this paper can effectively predict the driving characteristics of the SMA spring actuator and has theoretical guiding significance for the design of the SMA spring actuator.

## Figures and Tables

**Table 1:** Main parameters of deflection actuator.

SMA Spring		Bias Spring	
Density ρ (Kg/m^3^)	6500	Specific heat *c_p_* (J/Kg.°C)	320
Resistance R (Ω)	0.15	Thermal conductivity *h* (W/m^2^.°C)	120
Ambient temperature *T_∞_* (°C)	20	Martensite elastic modulus *D_M_* (Pa)	28 × 10^9^
Austenite elastic modulus *D_A_* (Pa)	75 × 10^9^	Poisson’s Ratio *µ_SMA_*	0.33
Martensite transformation start temperature *M_s_* (°C)	45	Martensite transformation end temperature *M_f_* (°C)	25
Austenite transformation start temperature *A_s_* (°C)	45	Austenite transformation end temperature *A_f_* (°C)	65
Material constant *C_M_* (Pa/°C)	10.3 × 10^6^	Material constant *C_A_* (Pa/°C)	10.3 × 10^6^
Coefficient of thermal expansion Θ (Pa/°C)	0. 55 × 10^6^	effective coil number *n*	9
Mean diameter of coil *D* (mm)	7.3	wire diameter *d* (mm)	1.3
Elastic coefficient (*k_b_*)	850 N/m	initial length (*l_b_*)	24 mm

## Data Availability

The data are made available through the corresponding authors’ emails.

## References

[1] Liu G., Zhang X., Chen X., He Y., Cheng L., Huo M., Yin J., Hao F., Chen S., Wang P. (2021). Additive manufacturing of structural materials. Mater. Sci. Eng. R Rep..

[2] Hu K.J., Rabenorosoa K., Ouisse M. (2021). A Review of SMA-Based Actuators for Bidirectional Rotational Motion: Application to Origami Robots. Front. Robot. AI.

[3] Ikuta K. Micro/miniature shape memory alloy actuator. Proceedings of the IEEE International Conference on Robotics and Automation.

[4] Tautzenberger P. (1990). Thermal actuators: A comparison of shape memory alloys with thermostatic bimetals and wax actuators. Eng. Asp. Shape Mem. Alloy..

[5] El-Atab N., Mishra R.B., Al-Modaf F., Joharji L., Alsharif A.A., Alamoudi H., Diaz M., Qaiser N., Hussain M.M. (2020). Soft actuators for soft robotic applications: A review. Adv. Intell. Syst..

[6] SEELECKE S., MULLER I. (2004). Shape memory alloy actuators in smart structures: Modeling and simulation. Appl. Mech. Rev..

[7] Ballew W., Seelecke S. (2019). Mesoscopic free energy as a framework for modeling shape memory alloys. J. Intell. Mater. Syst. Struct..

[8] Deng E., Tadesse Y. (2021). A Soft 3D-Printed Robotic Hand Actuated by Coiled SMA. Actuators.

[9] Weirich A., Kuhlenkötter B. (2019). Applicability of shape memory alloys in aircraft interiors. Actuators.

[10] Bhatt N., Soni S., Singla A. Mathematical Model of SMA Spring Actuator in a Miniature Flexible Tube Robot. Proceedings of the 4th International and 19th National Biennial Conferences on Machines and Mechanisms.

[11] Koh J.S. (2018). Design of shape memory alloy coil spring actuator for improving performance in cyclic actuation. Materials.

[12] Park S.J., Kim U., Park C.H. (2020). A novel fabric muscle based on shape memory alloy springs. Soft Robot..

[13] Roshan U., Amarasinghe R., Dayananda N. (2018). Design and fabrication of a minimally invasive surgical device with customized shape memory alloy spring actuator. J. Robot. Netw. Artif. Life.

[14] Sobrinho J.M., Emiliavaca A., Cunha M.F., Souto C.R., Silva S.A., Ries A. (2020). Experimental and numerical analyses of a rotary motor using shape memory alloy mini springs. Sens. Actuators A Phys..

[15] Pillai R., Murali G., Gopal M. (2018). Modeling and simulation of a shape memory alloy spring actuated flexible parallel manipulator. Procedia Comput. Sci..

[16] Muralidharan M., Brolin A., Mithun R., Patil R., Palani I.A. (2020). Investigations on bending characteristics of soft mesh structure using shape memory alloy spring towards bio-inspired robotic applications. Iran. J. Sci. Technol. Trans. Mech. Eng..

[17] Muralidharan M., Palani I.A. (2021). Development of subcarangiform bionic robotic fish propelled by shape memory alloy actuators. Def. Sci. J..

[18] Xiang C., Yang H., Sun Z., Xue B., Hao L., Rahoman M.A., Davis S. (2017). The design, hysteresis modeling and control of a novel SMA-fishing-line actuator. Smart Mater. Struct..

[19] Nguyen X.T., Calderón A.A., Rigo A., Joey Z.G., Pérez-Arancibia N.O. (2020). SMALLBug: A 30-mg crawling robot driven by a high-frequency flexible SMA microactuator. IEEE Robot. Autom. Lett..

[20] Bena R., Nguyen X.T., Calderon A.A., Rigo A., Perez-Arancibia N.O. (2021). SMARTI: A 60-mg Steerable Robot Driven by High-Frequency Shape Memory Alloy Actuation. IEEE Robot. Autom. Lett..

[21] Cortez-Vega R., Chairez I., Luviano-Juárez A., Feliu-Batlle V. (2018). A hybrid dynamic model of shape memory alloy spring actuators. Measurement.

[22] Gédouin P.A., Pino L., Chirani S.A., Calloch S., Delaleau E., Bourgeot J.M. (2019). R-phase shape memory alloy helical spring based actuators: Modeling and experiments. Sens. Actuators A Phys..

[23] Shakiba S., Ayati M., Yousefi-Koma A. (2020). Development of hybrid prandtl–ishlinskii and constitutive models for hysteresis of shape-memory-alloy-driven actuators. Robotica.

[24] Tanaka K., Kobayashi S., Sato Y. (1986). Thermomechanics of transformation pseudoelasticity and shape memory effect in alloys. Int. J. Plast..

[25] Liang C., Rogers C.A. (1997). One-dimensional thermomechanical constitutive relations for shape memory materials. J. Intell. Mater. Syst. Struct..

[26] Tobushi H., Tanaka K. (1991). Deformation of a shape memory alloy helical spring: Analysis based on stress-strain-temperature relation. JSME Int. J. Ser. 1 Solid Mech. Strength Mater..

[27] Liang C., Rogers C.A. (1997). Design of shape memory alloy springs with applications in vibration control. J. Intell. Mater. Syst. Struct..

[28] Aguiar R.A., Savi M.A., Pacheco P.M. (2010). Experimental and numerical investigations of shape memory alloy helical springs. Smart Mater. Struct..

[29] Paiva A., Savi M.A., Braga A.M.B., Pacheco P.M.C.L. (2005). A constitutive model for shape memory alloys considering tensile–compressive asymmetry and plasticity. Int. J. Solids Struct..

[30] Huang B., Lv H., Song Y. (2019). Numerical simulation and experimental study of a simplified force-displacement relationship in superelastic SMA helical springs. Sensors.

[31] Rizzello G., Naso D., Seelecke S. Hysteresis modeling in thermal shape memory alloy wire actuators: An irreversible port-Hamiltonian approach. Proceedings of the 58th IEEE Conference on Decision and Control (CDC).

[32] Ahn K.K., Kha N.B. (2007). Internal model control for shape memory alloy actuators using fuzzy based Preisach model. Sens. Actuators A Phys..

[33] Dutta S.M., Ghorbel F.H. (2005). Differential hysteresis modeling of a shape memory alloy wire actuator. IEEE/ASME Trans. Mechatron..

[34] Liang C., Rogers C.A. (1992). A multi-dimensional constitutive model for shape memory alloys. J. Eng. Math..

[35] Bekker A., Brinson L.C. (1998). Phase diagram based description of the hysteresis behavior of shape memory alloys. Acta Mater..

[36] Meng L., Bao W., Li F., Li H. (2021). Hysteresis compensation control of a dielectric elastomer vibration isolator. J. Low Freq. Noise Vib. Act. Control.

[37] Rizzello G., Ferrante F., Naso D., Seelecke S. (2017). Robust Interaction Control of a Dielectric Elastomer Actuator With Variable Stiffness. IEEE-Asme Trans. Mechatron..

[38] Shi Z., Tian J., Luo R., Zhao G., Wang T. (2018). Multifeedback Control of a Shape Memory Alloy Actuator and a Trial Application. IEEE Trans. Syst. Man Cybern.-Syst..

